# Association between the Change in Blood Pressure during Normotensive Pregnancy and the Development of Hypertension in Later Life

**DOI:** 10.31662/jmaj.2022-0102

**Published:** 2022-11-30

**Authors:** Kaori Iino, Tsuyoshi Higuchi, Kanji Tanaka, Shigeyuki Nakaji, Yoshihito Yokoyama, Hideki Mizunuma

**Affiliations:** 1 Department of Obstetrics and Gynecology, Hirosaki University Graduate School of Medicine, Hirosaki, Japan; 2 Division of Health Sciences, Department of Disability and Health, Hirosaki University Graduate School of Medicine, Hirosaki, Japan; 3 Department of Social Medicine, Hirosaki University School of Medicine, Hirosaki, Japan

**Keywords:** cardiovascular disease, hypertension, maternal complications, pre-eclampsia

## Abstract

**Introduction:**

Women who experience maternal complications, including pre-eclampsia, have a higher risk of cardiovascular disease development. Although the mechanism remains unclear, there is a hypothesis that pregnancy would be a stress test for cardiovascular disease. This study aimed to investigate whether changes in blood pressure during pregnancy would be associated with developing hypertension, which is a main risk of cardiovascular disease.

**Methods:**

We conducted a retrospective study by collecting Maternity Health Record Books from 735 middle-aged women. Of these, 520 women were selected based on our criteria. 138 were defined as the hypertensive group according to the criteria of receiving antihypertensive medications or blood pressures of >140/90 mmHg at the survey. The rest 382 were defined as the normotensive group. We compared the blood pressures of the hypertensive group with those of the normotensive group during pregnancy and postpartum. Then, 520 women were divided into quartiles (Q1-Q4) according to their blood pressures during pregnancy. After the changes in blood pressure for each gestational month relative to nonpregnant measurements were calculated, the changes in blood pressure were compared among the four groups. Additionally, the rate of developing hypertension was evaluated among the four groups.

**Results:**

The average age of the participants was 54.8 years (range: 40-85 years) at the time of the study and 25.9 years (range: 18-44 years) at delivery. There were significant differences in blood pressure during pregnancy between the hypertensive group and the normotensive group. Meanwhile, these two groups did not indicate any differences in blood pressure in postpartum. Higher mean blood pressure during pregnancy was associated with smaller changes in blood pressure during pregnancy. The rate of development of hypertension in each group of systolic blood pressure was 15.9% (Q1), 24.6% (Q2), 29.7% (Q3), and 29.7% (Q4). The rate of development of hypertension in each group of diastolic blood pressure (DBP) was 18.8% (Q1), 24.6% (Q2), 22.5% (Q3), and 34.1% (Q4).

**Conclusions:**

Changes in blood pressure during pregnancy are small in women who have a higher risk of hypertension. Levels of blood pressure during pregnancy may be reflected in individual stiffness of blood vessels by the burden of pregnancy. If so, levels of blood pressure would be used to facilitate highly cost-effective screening and interventions for women with a high risk of cardiovascular diseases.

## Introduction

Pregnancy causes dramatic changes in the hemodynamics and an approximately 50% increase in the cardiac output ^(1)^, both of which place significant stress on the mother. During normal pregnancy, the cardiovascular system develops reduced vascular resistance and increased arterial compliance ^(2)^, which allow for adequate oxygen and nutrient transfer to the fetus through the placental circulation. Generally, both systolic blood pressure (SBP) and diastolic blood pressure (DBP) start to decrease at an early stage of normal gestation, reach a nadir at 16-20 weeks, and gradually return to the pregestational level ^(2), (3)^. Satter et al. hypothesized that this burden of pregnancy is affected by the mother’s underlying vascular risk factors ^(4)^, and numerous epidemiological studies and several guidelines have indicated that women who experience maternal complications, such as pre-eclampsia, are at a higher risk of cardiovascular disease (CVD) development ^(5), (6), (7), (8), (9), (10), (11), (12), (13), (14), (15), (16), (17), (18)^.

If pregnancy is a stress test for CVD in later life, we can identify the individual risks of CVD during pregnancy. Although previous researchers have evaluated the association between maternal complications and CVD development, data on maternal complications are insufficient for all women to evaluate their own risks of CVD.

In our previous study, we hypothesized that women with a greater number of CVD risk factors would be less able to adapt to their physiological changes, allowing blood pressures during pregnancy to reflect the risk of CVD ^(19)^. Our results revealed retrospectively that a 10 mmHg increase in the averaged DBP during pregnancy was associated with a 1.70-fold increase in the risk of hypertension later in life. We also found that blood pressure during puerperium is not associated with the development of hypertension. These results suggest that blood pressure during pregnancy is associated with an individual’s underlying risk of CVD; nevertheless, the mechanism behind this association was not discussed in our previous study.

In this study, we hypothesized that changes in blood pressure during pregnancy are associated with the risk of CVD. To evaluate this hypothesis, we conducted a retrospective study among middle-aged women and investigated whether changes in blood pressure during pregnancy would be a risk of developing hypertension, which is a main risk of CVD, by collecting Maternity Health Record Books, which contain pregnancy records.

## Materials and Methods

### Study design and participants

This retrospective study evaluated data from the Iwaki Health Promotion Project, a longitudinal, observational study of adult men and women who live in the Iwaki area (Hirosaki, Aomori, Japan). The Iwaki cohort has been studied since 2005 and includes approximately 1,000 participants per year (corresponding to approximately 10% of all the people in the Iwaki area). Trained staff recorded the participants’ heights, weights, blood pressures, histories of tobacco and alcohol use, and histories of illnesses. The blood pressure was measured using standardized methods, while the body mass index (BMI) was calculated in kg/m^2^. The presence of hypertension was determined based on the usage of antihypertensive medications or high blood pressure (over 140/90 mmHg) at the time of the study.

We used data from the Maternity Health Record Books of middle-aged women. All pregnant Japanese women receive a Maternity Health Record Book that contains their medical history and demographic information, including their body weight, abdominal circumference, uterine fundus height, blood pressure, and the presence or absence of proteinuria, glycosuria, and edema; these data are recorded every 1-4 weeks depending on the gestational week. Attending physicians also record information regarding the neonate and the course of delivery. These records are occasionally used as a reliable information of health checkup for infants and vaccination, and many Japanese women keep these books for decades after delivery. Several previous studies have used similar techniques to obtain perinatal data ^(15), (19), (20)^.

**Figure 1.**
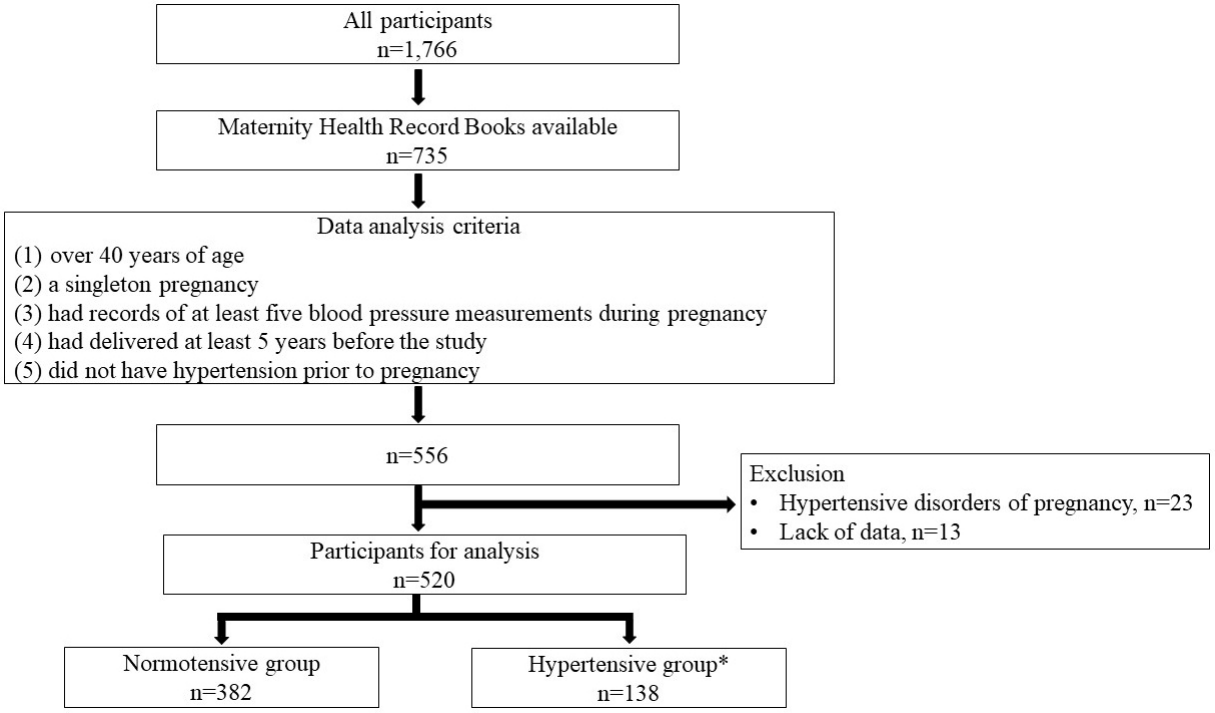
Flow diagram showing participant selection. *: Antihypertensive medication usage or blood pressures of ≥140/90 mmHg.

We collected 1,366 Maternity Health Record Books from 735 parous women between 2011 and 2015. From them, we selected 556 participants who (1) were over 40 years old, (2) had a singleton pregnancy, (3) had records of at least five blood pressure measurements during pregnancy, (4) had delivered at least 5 years before the study, and (5) did not have hypertension before pregnancy (Figure 1). In this study, we selected women over 40 years of age, because the prevalence of hypertension is very low in women younger than 40 years of age in Japan ^(21)^. We examined the first pregnancy of each participant, because it allowed for a longer observational period and because the incidence of hypertensive disorders of pregnancy (HDP) is the highest in the first pregnancy. Twenty-three women who developed HDP were excluded from the study, because HDP is a risk factor for hypertension later in life ^(13), (15)^. The diagnosis of HDP was based on the criteria of the Japan Society for the Study of Hypertension in Pregnancy (2018) ^(22)^. Thirteen women were further excluded due to a lack of data. Data from the first singleton pregnancy of 520 participants were included in the final analysis. Of the 520 participants, 138 were divided into the hypertensive group according to the criteria of receiving antihypertensive medications or blood pressures of ≥140/90 mmHg. The remaining 382 were defined as the normotensive group, that is, the control group.

This study was approved by the Hirosaki University Graduate School of Medicine Ethics Committee (Approval code; 2011-033, 2012-050, 2013-062, 2014-014, 2014-377-1) and was performed according to the 1964 Helsinki declaration and its later amendments or comparable ethical standards. All patients provided informed consent for their participation in this study and the publication of their data.

### Statistical analysis

Participants’ characteristics were compared between the hypertensive (developed hypertension later in life) group and the normotensive group using Student’s *t*-test and the chi-square test. The mean SBP/DBP values during gestational weeks 12-41 and in each trimester were obtained for each participant. Differences in the SBP/DBP in each gestational period and 1 month postpartum between the hypertensive and normotensive groups were also compared using Student’s *t*-test.

The mean values for SBP and DBP during gestational weeks 12-41 were obtained for each patient. The patients were grouped into quartiles (Q1-Q4) according to their mean SBP/DBP and the differences between blood pressures at 1 month postpartum and each gestational month were compared. We used data in 1 month postpartum as data in nonpregnant status at that time. Differences in blood pressure were converted into percentages, and the second, third, and fourth quartiles were compared with the first quartile using the Mann-Whitney U test.

All statistical analyses were performed using SPSS version 22 (IBM Inc., Armonk, NY, USA), and differences were considered statistically significant at p < 0.05.

## Results

Table 1 presents the demographic data and clinical characteristics of the participants. The overall mean age of the patients was 54.8 years (range: 40-85 years) at the time of the survey and 25.9 years (range: 18-44 years) at delivery. The mean follow-up period was 28.9 years (range: 5-52 years).

**Table 1.** Characteristics of Participants at Baseline and Delivery.

	Total N = 520	Normotensive N = 382	Hypertensive† N = 138	p
Follow-up period (years)	28.9 ± 10.6	26.7 ± 10.1	33.3 ± 8.9	<0.001^*^
Characteristics at enrollment				
Age (years)	54.8 ± 8.7	53.1 ± 8.0	58.3 ± 8.0	<0.001^*^
Body mass index (kg/m^2^)	25.9 ± 4.2	21.9 ± 2.9	24.3 ± 3.6	<0.001^*^
Menopause	349 (65.5%)	222 (58.1%)	115 (83.3%)	<0.001^**^
Smoking				0.437^**^
Never or former smoker	499 (93.6%)	327 (91.6%)	116 (94.3%)
Current smoker	34 (6.4%)	30 (8.4%)	7 (5.7%)
Alcohol consumption				
Yes	172 (32.3%)	135 (62.2%)	89 (72.4%)	<0.05^**^
No	361 (67.7%)	135 (37.8%)	34 (27.6%)	<0.05^**^
SBP (mmHg)	129.3 ± 17.9	118.8 ± 10.0	143.4 ± 16.0	<0.001^*^
DBP (mmHg)	77.9 ± 10.7	72.6 ± 7.8	85.8 ± 9.7	<0.001^*^
Delivery characteristics				
Age at delivery (years)	25.9 ± 4.2	26.3 ± 4.4	25.0 ± 3.6	0.002^*^
Gestational age ≥37 weeks	517 (97.0%)	370 (96.9%)	134 (97%)	0.867^**^
Birth weight ≥2,500 g	505 (94.7%)	361 (94.5%)	131 (95.0%)	0.734^**^

Abbreviations: systolic blood pressure (SBP); diastolic blood pressure (DBP) Values are presented as means ± standard deviations or n (%). †Antihypertensive medication usage or blood pressures of ≥140/90 mmHg. ^*^ Normotensive group vs Hypertensive group using Student’s *t*-test. ^**^ Normotensive group vs Hypertensive group using Chi-square test.

The mean age, BMI, and SBP/DBP were higher in the hypertensive group than in the normotensive group. The proportion of women at menopause was higher in the hypertensive group than in the normotensive group. These differences were consistent with the characteristics of hypertension. Alcohol consumption is a known risk factor for hypertension; nevertheless, in this study, the percentage of habitual alcohol drinkers was higher in the normotensive group than in the hypertensive group. Almost all participants had a normal course of pregnancy.

**Figure 2.**
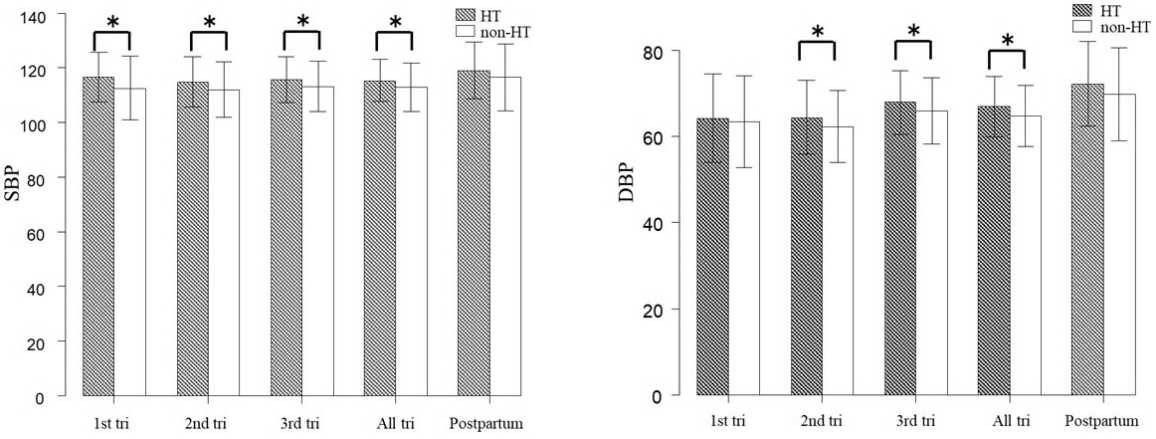
Differences in the mean values of SBP/DBP during pregnancy between women with HT and women without HT. Abbreviations: systolic blood pressure (SBP), diastolic blood pressure (DBP), hypertension (HT), and trimester (tri). The error bars indicate the standard deviation. *: significant differences (p < 0.05; Student’s t-test).

Figure 2 shows the differences in the mean SBP/DBP during pregnancy between the hypertensive group and the normotensive group. The mean SBP during the first trimester, second trimester, third trimester, and in all periods of pregnancy were significantly higher in the hypertensive group than in the normotensive group (p < 0.05 in all cases). The hypertensive group also showed significantly higher differences in the mean DBP during the second trimester, third trimester, and all periods of pregnancy (p < 0.05 in all cases). There were no differences in SBP/DBP between these two groups at postpartum.

Table 2 shows the mean SBP/DBP and characteristics of the four quartile groups. The follow-up period in DBP, current age, age at delivery, BMI, and current smoking status were not significantly different between the four groups. In the quartile groups according to SBP during pregnancy, there were significant differences in the follow-up period (Q1 vs Q3, Q1 vs Q4) and current age (Q1 vs Q4). Regarding SBP during pregnancy, the rate of developing hypertension was 15.9% in Q1, 24.6% in Q2, 29.7% in Q3, and 29.7% in Q4. AS for DBP, the rate of development of hypertension was 18.8% in Q1, 24.6% in Q2, 22.5% in Q3, and 34.1% in Q4. In both SBP and DBP during pregnancy, there were significant differences in the rate of hypertension among the four groups.

**Table 2.** Group Characteristics According to Mean Blood Pressure during Pregnancy.

	SBP during pregnancy					DBP during pregnancy				
	Q1 (N = 130)	Q2 (N = 130)	Q3 (N = 130)	Q4 (N = 130)	P-value	Q1 (N = 130)	Q2 (N = 130)	Q3 (N = 130)	Q4 (N = 130)	p-value
Follow-up period (years)	25.7 ± 10.1^a,b^	28.6 ± 10.4	29.4 ± 10.2^a^	30.3 ± 9.8^b^	0.002*	27.8 ± 9.8	28.0 ± 10.4	29.3 ± 10.0	28.8 ± 10.6	0.595^*^
Age (years)	52.5 ± 8.3^a^	54.8 ± 9.1	55.2 ± 7.3	55.3 ± 7.3^a^	0.022*	53.5 ± 8.1	54.3 ± 8.7	55.2 ± 8.5	54.9 ± 8.1	0.350^*^
Age at delivery (years)	26.8 ± 4.3	26.2 ± 4.0	25.9 ± 4.2^a^	25.0 ± 4.4^a^	0.005*	25.7 ± 4.0	26.3 ± 4.0	25.9 ± 4.4	26.1 ± 4.6	0.432^*^
Current BMI (kg/m^2^)	21.9 ± 3.6	22.7 ± 3.3	22.5 ± 2.7	23.0 ± 3.4	0.079*	22.2 ± 3.1	22.3 ± 3.6	22.6 ± 2.9	23.0 ± 3.6	0.242^*^
Current smoking	8 (6.8%)	10 (8.0%)	6 (5.2%)	13 (10.7%)	0.370**	9 (7.6%)	8 (6.8%)	6 (5.0%)	14 (11.5%)	0.483^**^
Hypertension† (%)	22 (15.9%)	34 (24.6%)	41 (29.7%)	41 (29.7%)	0.0189^**^	26 (18.8%)	34 (24.6%)	31 (22.5%)	47 (34.1%)	0.0255^**^

Abbreviations: systolic blood pressure (SBP); diastolic blood pressure (DBP); body mass index (BMI) †Antihypertensive medication usage or blood pressures ≥140/90 mmHg. *: One-way analysis of variance **: χ^2^ test a, b: p < 0.05 vs the first quartile (Q1) using Tukey’s test.

**Figure 3.**
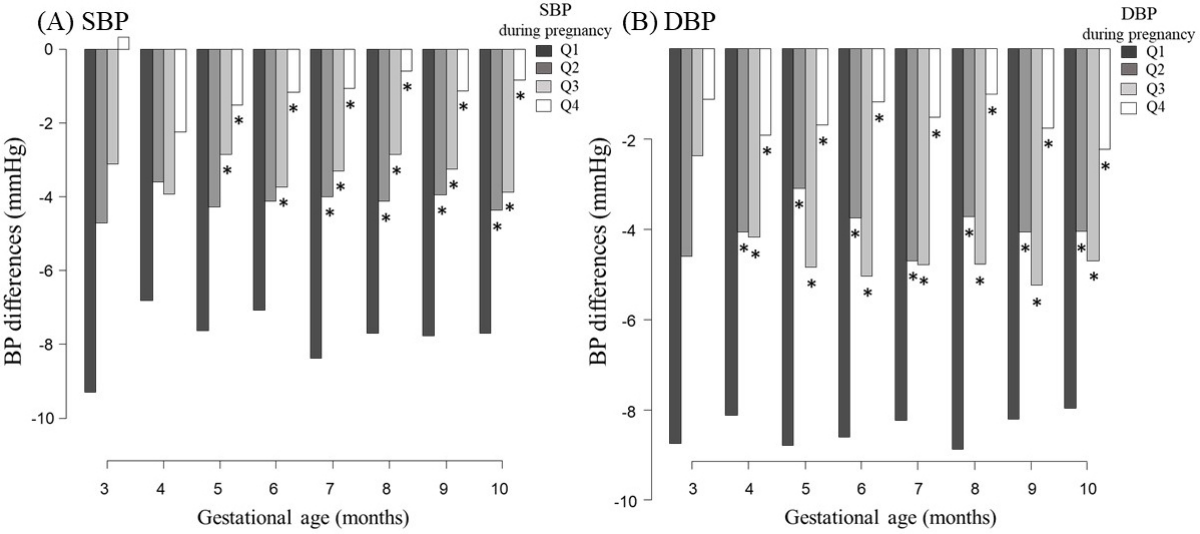
Changes in blood pressure for each gestational period relative to the nonpregnant measurements. Abbreviations: blood pressure (BP), systolic blood pressure (SBP), and diastolic blood pressure (DBP) *: p < 0.05 vs. the first quartile using the Mann-Whitney U test Participants were divided into quartiles (Q1-Q4) according to their blood pressures during pregnancy.

Figure 3 shows changes in the blood pressure for each gestational period relative to the nonpregnant measurements. Although blood pressures generally decreased during pregnancy, the magnitude of the decrease varied according to the blood pressure quartile. The first quartile group had the lowest blood pressure during pregnancy and exhibited the greatest decrease during pregnancy. By contrast, the fourth quartile group had the highest blood pressure but the smallest change during pregnancy. Similar trends were observed for SBP and DBP.

## Discussion

This study is the first to investigate whether changes in blood pressure during pregnancy would be a risk of developing hypertension. We found that blood pressure changes during pregnancy varied between individuals, and these differences were associated with the risk of developing hypertension.

CVD accounts for 30% of deaths worldwide and is associated with heavy medical and economic burdens ^(23)^. Thus, highly cost-effective measures are needed to prevent and treat CVD. Many previous reports indicated the possibility that pregnancy itself would be a useful stress test for CVD ^(9), (11), (12), (14), (17), (18), (24), (25)^. If pregnancy itself is used as a stress test for CVD, even normal pregnancy could expose the underlying risks of CVD. Nevertheless, previous studies have not discussed this theme in detail. Our previous research revealed that DBP during normotensive pregnancy was statistically associated with the risk of developing hypertension ^(19)^. In this study, we compared the changes in blood pressure during pregnancy between four quartiles divided by the mean value of blood pressure during pregnancy. Our results revealed that higher mean blood pressure during pregnancy was associated with smaller changes in blood pressure during pregnancy. Additionally, women whose blood pressures were high showed an apparent tendency to develop hypertension. We also showed that there were significant differences in blood pressure during pregnancy between the current hypertensive group and the normotensive group. However, in the nonpregnant status at that time, these two groups did not indicate any differences in blood pressure. These findings might indicate that women who have stiffened blood vessels are unable to have sufficient reduced levels of blood pressure during pregnancy. If this hypothesis is true, the information regarding blood pressure during pregnancy would be a useful predictive marker for the development of hypertension.

This study had three limitations. First, we only evaluated data from women with Maternity Health Record Books. Approximately 30% of parous women brought these books, which may have resulted in selection bias. Second, participation in the Iwaki Health Promotion Project is voluntary, and the project’s participants may represent a subset of healthy patients with a low incidence of hypertension. To avoid selection bias, a longitudinal study using hospital records is needed in the future. Third, the participants of this study were from a rural area in Japan. Hence, it may not be possible to generalize our findings to other populations.

In conclusion, we found that pregnant women with high blood pressure showed a small decrease in blood pressure during pregnancy and that these women developed hypertension at a high rate. These results can be used to facilitate highly cost-effective screening and interventions for women with a high risk of CVD. However, our study was small-scale and did not include basic research. Therefore, to elucidate the association between pregnancy and the risk of CVD, further studies are required.

## Article Information

Hideki Mizunuma Deceased July 9, 2020.

### Conflicts of Interest

None

### Sources of Funding

This work was supported by KAKENHI (18K16753), Japan Society for the Promotion of Science (JSPS).

### Author Contributions

H. Mizunuma and K. Iino designed this study. T. Higuchi, H. Mizunuma, K. Tanaka, Y. Yokoyama, and K. Iino interpreted data. K. Iino collected and analyzed data. K. Iino and H. Mizunuma wrote the report. H. Mizunuma and S. Nakaji managed this study.

### Approval by Institutional Review Board (IRB)

This study was approved by the Hirosaki University Graduate School of Medicine Ethics Committee. Approval code; 2011-033, 2012-050, 2013-062, 2014-014, 2014-377-1.
